# Construction of a risk prediction model for pulmonary infection in patients with spontaneous intracerebral hemorrhage during the recovery phase based on machine learning

**DOI:** 10.3389/fneur.2025.1571755

**Published:** 2025-06-18

**Authors:** Jixiang Xu, Yuan Li, Fumin Zhu, Xiaoxiao Han, Liang Chen, Yinliang Qi, Xiaomei Zhou

**Affiliations:** ^1^Department of Hyperbaric Oxygen, The Second People's Hospital of Hefei, Hefei Hospital Affiliated to Anhui Medical University, Hefei, Anhui Province, China; ^2^Department of Neurology, Dazhou Central Hospital, Dazhou, Sichuan, China; ^3^Wannan Medical College, Wuhu, Anhui, China; ^4^Anhui Medical University, Hefei, Anhui, China

**Keywords:** pulmonary infection, deep subcortical intracerebral hemorrhage, machine learning algorithms, prediction model, SHAP analysis

## Abstract

**Objective:**

Pulmonary infection (PI) remains a prevalent and severe complication in patients recovering from spontaneous deep subcortical intracerebral hemorrhage (deep SICH). Accurate prediction of PI risk is crucial for early intervention and optimized clinical management. The aim of this study was to develop a machine learning (ML) model for predicting PI risk in patients during the recovery phase of deep SICH and to investigate the contributions of individual risk factors through explainable artificial intelligence techniques.

**Methods:**

We conducted a retrospective study involving 649 patients diagnosed with PI during the recovery phase of deep SICH between 2021 and 2023. The cohort was divided into a training set (70%, *n* = 454) and a testing set (30%, *n* = 195). Eight key clinical features were identified using the Boruta algorithm: mechanical ventilation, nasogastric feeding, tracheotomy, antibacterial drug use, hyperbaric oxygen therapy, procalcitonin levels, sedative drug use, and consciousness scores. Seven ML algorithms were employed to build predictive models, with performance evaluated based on the area under the receiver operating characteristic (AUC) curve, sensitivity, specificity, and accuracy. The best-performing model was selected, and SHAP (Shapley Additive Explanations) analysis was performed to interpret feature importance.

**Results:**

Among 649 patients with deep SICH, no significant baseline differences were found between the training (*n* = 454) and testing (*n* = 195) sets. The Boruta algorithm identified eight key predictors of pulmonary infection (PI). The random forest (RF) model achieved the highest AUCs: 0.994 (95% CI: 0.989–0.998) in training and 0.931 (95% CI: 0.899–0.963) in testing. DeLong tests showed RF significantly outperformed several models (DT, SVM, LightGBM), while performance differences with XGBoost (*p* = 0.95), KNN (*p* = 0.80), and LR (*p* = 0.22) were not significant. SHAP analysis revealed mechanical ventilation, nasogastric feeding, and tracheotomy as key risk factors, with hyperbaric oxygen therapy and higher consciousness scores showing protective effects.

**Conclusions:**

This study provides a high-performing and interpretable ML-based risk stratification tool for pulmonary infection in patients during the recovery phase of deep SICH. The integration of SHAP enhances clinical applicability by demystifying complex model outputs, thereby supporting individualized preventive strategies. These findings underscore the promise of explainable AI in advancing neurocritical care and call for prospective multicenter validation and real-time dynamic model adaptation in future research.

## Background

Spontaneous intracerebral hemorrhage (SICH) persists as one of the deadliest and most debilitating subtypes of cerebrovascular disease worldwide (1, 2). Despite considerable advancements in surgical techniques and critical care management, up to 50% of patients succumb within 30 days following the onset of SICH (3). For those who survive, the stabilization of their condition marks the commencement of a crucial recovery phase. Although there is no universally standardized definition of the recovery phase in SICH, mounting evidence and clinical experience suggest that the subacute to early chronic stage—ranging from 2 weeks to 6 months post-onset—represents a critical period for functional recovery and rehabilitation. The ESO guidelines recommend initiating rehabilitation within 24 to 48 h after SICH onset, though generally not before 24 h, and usually after clinical stabilization, which occurs by the 2 month (4). Saulle et al. (5) highlighted that clinical research specifically targeting the recovery phase remains scarce and lacks a uniform time definition. Liu et al. (6) demonstrated that initiating rehabilitation ~1 week after onset significantly reduces 6-month mortality and hospitalization duration. Notably, Cao et al. (7) defined the recovery phase in SICH patients as the period between 2 and 6 months post-onset. Kearns et al. (8) further reported that the interval from ~72 h to 14 days post-onset represents a crucial stage of hematoma resolution, inflammation attenuation, and early neurofunctional recovery, thereby supporting the rationale for selecting 2 weeks as a pragmatic threshold for defining the onset of the recovery phase. This definition aligns well with our clinical observations, wherein neurological deficits tend to stabilize and the demand for structured rehabilitation intensifies during this time frame. Drawing upon this converging body of evidence, we pragmatically define the recovery phase in the present study as the period extending from 2 weeks to 6 months following SICH onset.

Anatomically, SICH can be classified into lobar hemorrhage and deep subcortical hemorrhage. Previous research has demonstrated that lobar cerebral hemorrhage is typically associated with a more severe early prognosis and is mainly caused by non-hypertensive mechanisms, such as cerebral amyloid angiopathy, which poses more complex clinical challenges. In contrast, deep subcortical hemorrhage is often attributed to hypertensive causes and is associated with lower early mortality; however, patients remain susceptible to multiple complications during the recovery phase, including pulmonary infection (PI) (9). A meta-analysis of 130,000 post-stroke infection cases found that ~10% of SICH patients in the recovery phase develop PI (10), which increases mortality by ~30% (11, 12). Most existing studies on risk factors (13) for PI and clinical prediction models (14, 15) have predominantly focused on the acute phase of SICH, with limited attention given to the recovery phase. The physiological state of patients during recovery differs markedly from that of the acute phase and represents a critical window for functional restoration. Patients with deep subcortical hemorrhage are often bedridden for extended periods and may experience immunosuppression, making them particularly vulnerable to infections (16). These factors underscore an urgent clinical need for a dedicated risk stratification tool to predict PI specifically in deep SICH patients during the recovery phase.

Machine learning, a technology capable of identifying and learning patterns from large datasets, has shown significant potential in predicting diseases and treatment outcomes within the medical field (17, 18). Compared to traditional statistical models, machine learning methods excel in capturing complex non-linear relationships (19). This study focuses on patients with deep subcortical hemorrhage during the recovery phase and aims to develop a predictive model for PI using several ML algorithms, including logistic regression, random forest, decision tree, k-nearest neighbors, light gradient boosting machine, support vector machine, and extreme gradient boosting. The performance of each model will be evaluated, and the optimal model will be interpreted using SHapley Additive exPlanations (SHAP) (20). Importantly, the goal of this study is not to predict mortality or long-term functional outcomes, but rather to enable early identification of patients at high risk of PI. This facilitates proactive intervention and personalized care strategies during a critical window of neurological recovery. Given the distinct clinical characteristics and complication mechanisms of deep subcortical hemorrhage compared to lobar hemorrhage during recovery, this study offers important value in constructing a targeted prediction model for this specific patient population.

## Materials and methods

### Study design and patient selection

**Figure 1:**
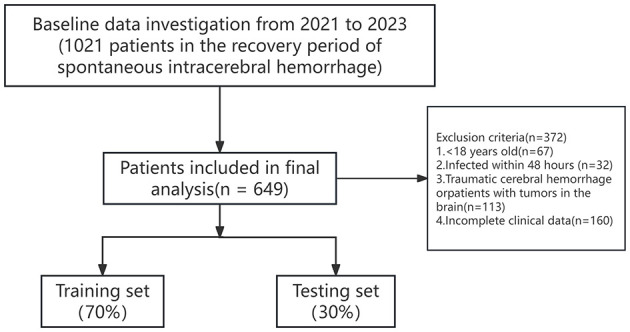
Flowchart of the process of patient enrollment. Patients with other severe neurological disorders (e.g., Parkinson's disease, Alzheimer's disease, refractory epilepsy) or systemic comorbidities (e.g., end-stage renal disease, advanced COPD, metastatic cancer) were also excluded to ensure population homogeneity. No patients were excluded due to in-hospital or follow-up death. All 649 patients completed the study period without mortality.

The study population consisted of 1,021 patients diagnosed with deep SICH and admitted to the Second People's Hospital of Hefei, Anhui Province, China, between January 2021 and December 2023. The inclusion criteria were: (1) diagnosis of deep SICH with confirmation of entering the recovery phase (21); (2) age ≥ 18 years; (3) complete clinical and follow-up data available. The exclusion criteria were: (1) presence of other severe neurological disorders or comorbidities, including but not limited to neurodegenerative diseases (e.g., Parkinson's disease, Alzheimer's disease), intracranial space-occupying lesions (e.g., brain tumors), epilepsy with recurrent seizures, or severe systemic conditions such as end-stage renal disease, advanced chronic obstructive pulmonary disease (COPD), or malignancies with systemic metastasis; (2) incomplete data or loss to follow-up (The initial dataset included 1,021 patients extracted from the hospital information system (HIS). During preprocessing, patients with incomplete clinical records were excluded based on predefined criteria. All included variables were assessed for missing data using SPSS frequency analysis, and no missing values were detected in the final dataset). Ultimately, 649 patients were included in the analysis, and the flow chart of the selection process is presented in Figure 1.

The 649 patients included in the study were sequentially numbered based on their admission dates and defined as the overall cohort dataset. Using the “sample()” function in R, the overall cohort dataset was randomly divided into training and testing set in a 7:3 ratio, comprising 454 patients in the training set and 195 patients in testing set. T This retrospective study was conducted using previously collected electronic medical records at Hefei Second People's Hospital. The study protocol was reviewed and approved by the Ethics Committee of Hefei Second People's Hospital (Ethics Number: 2022-Scientific Research-091). The requirement for informed consent was formally waived by the Ethics Committee, as the study involved no more than minimal risk to the participants, used fully de-identified data, and did not affect patient rights or welfare.

### Data extraction

In this study, the selection of variables was systematically informed by clinical relevance, evidence-based literature, expert consensus, and the cumulative experience of our multidisciplinary research team. The selection process prioritized variables with plausible associations to the study's primary outcome—namely, the onset and progression of pulmonary infection (PI) in the post-ICH recovery context. The inclusion criteria for candidate variables were delineated as follows:

#### Clinical relevance

Variables with established significance in clinical practice were given priority. For instance, types of intracerebral hemorrhage (e.g., basal ganglia hemorrhage, brainstem hemorrhage, intraventricular hemorrhage, cerebellar hemorrhage, and thalamic hemorrhage) were included because of their established impact on patient prognosis and their potential to cause secondary complications, including pulmonary infections.

#### Previous studies

Variables identified as risk factors for PI or outcomes associated with intracerebral hemorrhage in prior research were incorporated. These variables included age, gender, smoking history, and drinking history (15, 22–24).

#### Biochemical indicators

Biochemical indicators obtained from the most recent laboratory tests conducted closest in time to the diagnosis of PI were selected for their diagnostic value in identifying and monitoring infection progression. The indicators included white blood cell count (WBC), absolute lymphocyte count (ALC), neutrophil percentage (NE%), hemoglobin (Hb), platelet count (PLT), total bilirubin (TBIL), direct bilirubin (DBIL), indirect bilirubin (IBIL), alanine aminotransferase (ALT), aspartate aminotransferase (AST), prealbumin (PAB), albumin (ALB), blood urea nitrogen (BUN), creatinine (Cr), serum potassium (K^+^), C-reactive protein (CRP), serum amyloid A (SAA), procalcitonin (PCT), lactate dehydrogenase (LDH), triglycerides (TG), total cholesterol (TC), prothrombin time (PT), activated partial thromboplastin time (APTT), and D-dimer (D-D).

#### Intervention-related variables

Variables associated with the provided interventions were included to evaluate their influence on PI outcomes, including the use of broad-spectrum antibiotics, hyperbaric oxygen therapy (HBOT), mechanical ventilation, vasoactive drugs, sedatives, analgesics, and anticoagulants.

#### Surgical and procedural factors

Factors associated with an increased risk of PI, such as invasive procedures and tracheotomy, were included.

#### Functional status assessment

The Barthel Index (BI), which measures the activities of daily living, along with variables such as consciousness status score (Glasgow Coma Scale, GCS) and dysphagia score (Standardized Swallowing Assessment, SSA), were selected to evaluate their relationship with the overall functional status of patients and the likelihood of developing PI.

#### Hospitalization data

Length of hospital stay and number of hospitalizations were included to understand the impact of prolonged or repeated hospitalizations on the risk of PI.

### Feature selection and machine learning

This study utilizes the Boruta algorithm to identify key features associated with the risk of PI in patients with deep SICH. The Boruta algorithm (25) is a feature selection method built on the random forest (RF) algorithm. During its application, each original feature is paired with a corresponding shadow feature, which is generated by randomly shuffling the values of the original feature. Both the original and shadow features are utilized as inputs to train the RF model, and importance scores are calculated for each feature. The Boruta algorithm compares the importance scores of the original features with those of the shadow features to identify features that demonstrate significantly higher importance than their shadow counterparts. Only features with substantially higher importance scores than their shadow counterparts are deemed significant and retained in the final feature set. After feature selection, multiple machine learning algorithms were employed to construct a risk prediction model for aspiration, including Logistic Regression (LR), Random Forest (RF), Decision Tree (DT), k-Nearest Neighbors (k-NN), Light Gradient Boosting Machine (LightGBM), Support Vector Machine (SVM), and Extreme Gradient Boosting (XGBoost). Each of these algorithms has unique advantages: LR is mainly used for predicting categorical outcomes based on specific features; RF enhances prediction accuracy by aggregating multiple decision trees through majority voting; DT creates an interpretable tree structure by splitting attributes; k-NN is an instance-based learning approach suitable for scenarios without explicit model training; LightGBM is optimized for efficiently processing large-scale datasets; SVM classifies by maximizing the margin between classes, making it suitable for high-dimensional data; and XGBoost improves predictive performance by iteratively building decision trees and minimizing the loss function.

### Model construction and evaluation

In this study, the LR model was developed using the “glm” function, the RF model was constructed using the “randomForest” package, and the Decision Tree model was implemented using the “rpart” package. For k-NN, the “knn” function from the “class” package was implemented using; the LightGBM model was developed with the “lightgbm” package, the SVM model was built using the “svm” function from the “e1071” package, and the XGBoost model was constructed with the “xgboost” package. Each model was trained exclusively within the training dataset using 10-fold cross-validation repeated 5 times. This approach ensured robust internal validation and minimized overfitting. No hyperparameter tuning was performed; models used default or empirically defined settings. Final evaluation was conducted on the independent test set. Pairwise AUC comparisons between models were performed using the Delong test, implemented via the “pROC” package in R.

The evaluation metrics for the models include accuracy (ACC), sensitivity (SEN), specificity (SPE), positive predictive value (PPV), negative predictive value (NPV), and area under the receiver operating characteristic curve (AUC). Additionally, the Shapley Additive Explanations (SHAP) method was applied to further elucidate the contribution of each feature variable to the models (26). SHAP plots visualize the positive and negative contributions of each feature to the model's predictions, enabling the identification of features with significant influence on the prediction of PI risk.

### Statistical analysis

Statistical analyses were performed using SPSS 26.0 and R 4.3.3 software. The continuous variables were statistically analyzed by *t*-test (normal distribution data) or *M*-*U* test (non-normal distribution data). The normal distribution data were represented by mean ± standard deviation, and the non-normal distribution data were represented by quartiles. The categorical data were analyzed by a Chi-square test or Fisher precision test, and were displayed as percentage. When *P* < 0.05 (bilateral), the difference was considered to be significant.

## Materials and methods

### Study design and patient selection

The study population consisted of 1,021 patients diagnosed with deep SICH and admitted to the Second People's Hospital of Hefei, Anhui Province, China, between January 2021 and December 2023. The inclusion criteria were: (1) diagnosis of deep SICH with confirmation of entering the recovery phase (21); (2) age ≥18 years; (3) complete clinical and follow-up data available. The exclusion criteria were: (1) presence of other severe neurological disorders or comorbidities, including but not limited to neurodegenerative diseases (e.g., Parkinson's disease, Alzheimer's disease), intracranial space-occupying lesions (e.g., brain tumors), epilepsy with recurrent seizures, or severe systemic conditions such as end-stage renal disease, advanced chronic obstructive pulmonary disease (COPD), or malignancies with systemic metastasis; (2) incomplete data or loss to follow-up (The initial dataset included 1,021 patients extracted from the hospital information system (HIS). During preprocessing, patients with incomplete clinical records were excluded based on predefined criteria. All included variables were assessed for missing data using SPSS frequency analysis, and no missing values were detected in the final dataset). Ultimately, 649 patients were included in the analysis, and the flow chart of the selection process is presented in Figure 1.

The 649 patients included in the study were sequentially numbered based on their admission dates and defined as the overall cohort dataset. Using the “sample()” function in R, the overall cohort dataset was randomly divided into training and testing set in a 7:3 ratio, comprising 454 patients in the training set and 195 patients in testing set. T This retrospective study was conducted using previously collected electronic medical records at Hefei Second People's Hospital. The study protocol was reviewed and approved by the Ethics Committee of Hefei Second People's Hospital (Ethics Number: 2022-Scientific Research-091). The requirement for informed consent was formally waived by the Ethics Committee, as the study involved no more than minimal risk to the participants, used fully de-identified data, and did not affect patient rights or welfare.

### Data extraction

In this study, the selection of variables was systematically informed by clinical relevance, evidence-based literature, expert consensus, and the cumulative experience of our multidisciplinary research team. The selection process prioritized variables with plausible associations to the study's primary outcome—namely, the onset and progression of pulmonary infection (PI) in the post-ICH recovery context. The inclusion criteria for candidate variables were delineated as follows:

#### Clinical relevance

Variables with established significance in clinical practice were given priority. For instance, types of intracerebral hemorrhage (e.g., basal ganglia hemorrhage, brainstem hemorrhage, intraventricular hemorrhage, cerebellar hemorrhage, and thalamic hemorrhage) were included because of their established impact on patient prognosis and their potential to cause secondary complications, including pulmonary infections.

#### Previous studies

Variables identified as risk factors for PI or outcomes associated with intracerebral hemorrhage in prior research were incorporated. These variables included age, gender, smoking history, and drinking history (15, 22–24).

#### Biochemical indicators

Biochemical indicators obtained from the most recent laboratory tests conducted closest in time to the diagnosis of PI were selected for their diagnostic value in identifying and monitoring infection progression. The indicators included white blood cell count (WBC), absolute lymphocyte count (ALC), neutrophil percentage (NE%), hemoglobin (Hb), platelet count (PLT), total bilirubin (TBIL), direct bilirubin (DBIL), indirect bilirubin (IBIL), alanine aminotransferase (ALT), aspartate aminotransferase (AST), prealbumin (PAB), albumin (ALB), blood urea nitrogen (BUN), creatinine (Cr), serum potassium (K^+^), C-reactive protein (CRP), serum amyloid A (SAA), procalcitonin (PCT), lactate dehydrogenase (LDH), triglycerides (TG), total cholesterol (TC), prothrombin time (PT), activated partial thromboplastin time (APTT), and D-dimer (D-D).

#### Intervention-related variables

Variables associated with the provided interventions were included to evaluate their influence on PI outcomes, including the use of broad-spectrum antibiotics, hyperbaric oxygen therapy (HBOT), mechanical ventilation, vasoactive drugs, sedatives, analgesics, and anticoagulants.

#### Surgical and procedural factors

Factors associated with an increased risk of PI, such as invasive procedures and tracheotomy, were included.

#### Functional status assessment

The Barthel Index (BI), which measures the activities of daily living, along with variables such as consciousness status score (Glasgow Coma Scale, GCS) and dysphagia score (Standardized Swallowing Assessment, SSA), were selected to evaluate their relationship with the overall functional status of patients and the likelihood of developing PI.

#### Hospitalization data

Length of hospital stay and number of hospitalizations were included to understand the impact of prolonged or repeated hospitalizations on the risk of PI.

### Feature selection and machine learning

This study utilizes the Boruta algorithm to identify key features associated with the risk of PI in patients with deep SICH. The Boruta algorithm (25) is a feature selection method built on the random forest (RF) algorithm. During its application, each original feature is paired with a corresponding shadow feature, which is generated by randomly shuffling the values of the original feature. Both the original and shadow features are utilized as inputs to train the RF model, and importance scores are calculated for each feature. The Boruta algorithm compares the importance scores of the original features with those of the shadow features to identify features that demonstrate significantly higher importance than their shadow counterparts. Only features with substantially higher importance scores than their shadow counterparts are deemed significant and retained in the final feature set. After feature selection, multiple machine learning algorithms were employed to construct a risk prediction model for aspiration, including Logistic Regression (LR), Random Forest (RF), Decision Tree (DT), k-Nearest Neighbors (k-NN), Light Gradient Boosting Machine (LightGBM), Support Vector Machine (SVM), and Extreme Gradient Boosting (XGBoost). Each of these algorithms has unique advantages: LR is mainly used for predicting categorical outcomes based on specific features; RF enhances prediction accuracy by aggregating multiple decision trees through majority voting; DT creates an interpretable tree structure by splitting attributes; k-NN is an instance-based learning approach suitable for scenarios without explicit model training; LightGBM is optimized for efficiently processing large-scale datasets; SVM classifies by maximizing the margin between classes, making it suitable for high-dimensional data; and XGBoost improves predictive performance by iteratively building decision trees and minimizing the loss function.

### Model construction and evaluation

In this study, the LR model was developed using the “glm” function, the RF model was constructed using the “randomForest” package, and the Decision Tree model was implemented using the “rpart” package. For k-NN, the “knn” function from the “class” package was implemented using; the LightGBM model was developed with the “lightgbm” package, the SVM model was built using the “svm” function from the “e1071” package, and the XGBoost model was constructed with the “xgboost” package. Each model was trained exclusively within the training dataset using 10-fold cross-validation repeated 5 times. This approach ensured robust internal validation and minimized overfitting. No hyperparameter tuning was performed; models used default or empirically defined settings. Final evaluation was conducted on the independent test set. Pairwise AUC comparisons between models were performed using the Delong test, implemented via the “pROC” package in R.

The evaluation metrics for the models include accuracy (ACC), sensitivity (SEN), specificity (SPE), positive predictive value (PPV), negative predictive value (NPV), and area under the receiver operating characteristic curve (AUC). Additionally, the Shapley Additive Explanations (SHAP) method was applied to further elucidate the contribution of each feature variable to the models (26). SHAP plots visualize the positive and negative contributions of each feature to the model's predictions, enabling the identification of features with significant influence on the prediction of PI risk.

### Statistical analysis

Statistical analyses were performed using SPSS 26.0 and R 4.3.3 software. The continuous variables were statistically analyzed by *t* test (normal distribution data) or *M*-*U* test (non-normal distribution data). The normal distribution data were represented by mean ± standard deviation, and the non-normal distribution data were represented by quartiles. The categorical data were analyzed by a Chi-square test or Fisher precision test, and were displayed as percentage. When *P* < 0.05 (bilateral), the difference was considered to be significant. This study was conducted and reported in accordance with the TRIPOD (Transparent Reporting of a multivariable prediction model for Individual Prognosis Or Diagnosis) guidelines.

## Results

### Baseline patient characteristics

This study included 649 patients in the recovery phase of deep SICH, comprising 454 patients in the training set (301 with PI and 153 without PI) and 195 patients in the testing set (116 with PI and 79 without PI). The baseline characteristics of the patients included demographic data, clinical interventions, biochemical markers, and functional status evaluations. No statistically significant differences (*P* > 0.05) were observed in the baseline characteristics between the training and testing set, indicating that the two sets were balanced in terms of baseline characteristics. The detailed baseline data are presented in Table 1.

**Table 1:** Patient demographics and baseline characteristics.

**Variables**	**Total (*n =* 649)**	**Train (*n =* 454)**	**Test (*n =* 195)**	***p***
Age, years	59 (52, 70)	60 (53, 70)	58 (51, 68.5)	0.057
**Gender**, ***n*** **(%)**				0.712
female	275 (42)	195 (43)	80 (41)	
male	374 (58)	259 (57)	115 (59)	
**Smoking**, ***n*** **(%)**				0.672
no	409 (63)	289 (64)	120 (62)	
yes	240 (37)	165 (36)	75 (38)	
**Drinking**, ***n*** **(%)**				0.699
no	405 (62)	286 (63)	119 (61)	
yes	244 (38)	168 (37)	76 (39)	
**Type of intracerebral hemorrhage**, ***n*** **(%)**				0.461
Basal Ganglia Hemorrhage	259 (40)	180 (40)	79 (41)	
Brainstem hemorrhage	87 (13)	58 (13)	29 (15)	
Ventricular hemorrhage	61 (9)	46 (10)	15 (8)	
Cerebellar hemorrhage	194 (30)	132 (29)	62 (32)	
Thalamic hemorrhage	48 (7)	38 (8)	10 (5)	
**Antibacterial**, ***n*** **(%)**				0.321
no	259 (40)	175 (39)	84 (43)	
yes	390 (60)	279 (61)	111 (57)	
**Hyperbaric oxygen**, ***n*** **(%)**				0.313
no	387 (60)	277 (61)	110 (56)	
yes	262 (40)	177 (39)	85 (44)	
**Mechanical ventilation**, ***n*** **(%)**				0.785
no	276 (43)	191 (42)	85 (44)	
yes	373 (57)	263 (58)	110 (56)	
**Vasoactive drugs**, ***n*** **(%)**				0.913
no	429 (66)	299 (66)	130 (67)	
yes	220 (34)	155 (34)	65 (33)	
**Sedative drugs**, ***n*** **(%)**				0.214
no	327 (50)	221 (49)	106 (54)	
yes	322 (50)	233 (51)	89 (46)	
**Analgesic drugs**, ***n*** **(%)**				0.153
no	459 (71)	313 (69)	146 (75)	
yes	190 (29)	141 (31)	49 (25)	
**Anticoagulant drugs**, ***n*** **(%)**				0.34
no	343 (53)	246 (54)	97 (50)	
yes	306 (47)	208 (46)	98 (50)	
WBC, × 10^9^/L	8.88 (6.26, 14.7)	9.38 (6.34, 14.91)	8.29 (6.12, 13.1)	0.147
ALC, × 10^9^/L	1.85 (1.33, 4.19)	1.81 (1.32, 4.17)	1.89 (1.33, 4.22)	0.819
NE, %	68.3 (60, 74.8)	68.45 (60, 74.97)	68.1 (60.2, 74.7)	0.536
HB, g/L	111 (97, 122)	111 (97, 123)	110 (93.5, 121)	0.23
PLT, × 10^9^/L	256 (198, 325)	263.5 (202, 328)	242 (191, 311)	0.075
TBIL, μmol/L	9.8 (7.5, 12.7)	9.8 (7.43, 12.67)	10 (7.6, 12.7)	0.96
DBIL, μmol/L	2.9 (2.2, 3.9)	2.8 (2.2, 3.9)	3 (2.1, 3.9)	0.873
IBIL, μmol/L	6.7 (5, 9)	6.7 (5, 9.07)	6.6 (4.9, 8.85)	0.65
ALT, U/L	21 (12, 34)	21 (12.25, 35)	21 (12, 31.5)	0.542
AST, U/L	24 (17, 33)	24 (17, 32.75)	22 (15.5, 32.5)	0.143
PAB, mg/L	193.8 (153.6, 247)	191.2 (153.02, 241.38)	202.8 (157.3, 251.35)	0.211
ALB, g/L	34.2 (31.8, 36.6)	34.1 (31.7, 36.5)	34.3 (32.1, 37.05)	0.329
BUN, mmol/L	4.57 (3.53, 6.09)	4.57 (3.43, 6.09)	4.6 (3.72, 6.1)	0.862
Cr, μmol/L	45 (34.2, 58)	44.45 (33.82, 57.4)	46.2 (35.4, 60.9)	0.428
K+, mmol/L	4.04 (3.75, 4.29)	4.05 (3.72, 4.29)	4.04 (3.8, 4.3)	0.474
CRP, mg/L	15.28 (6.11, 30.63)	15.42 (6.39, 30.57)	14.5 (5.08, 29.9)	0.367
SAA, mg/L	81 (17.4, 94.8)	81 (19.52, 98.3)	77.2 (14.45, 91.9)	0.092
PCT, ng/mL	0.43 (0.43, 0.7)	0.43 (0.43, 0.72)	0.43 (0.43, 0.65)	0.682
LDH, U/L	203 (165, 251)	211.5 (170, 258)	188 (158, 238)	0.016
TG, mmol/L	1.33 (1.04, 1.69)	1.3 (1.04, 1.68)	1.42 (1.03, 1.73)	0.254
TC, mmol/L	3.83 (3.25, 4.29)	3.83 (3.28, 4.29)	3.84 (3.14, 4.31)	0.491
PT, S	13.4 (12.9, 14)	13.2 (12.7, 14.1)	13.5 (12.3, 14.5)	0.921
APTT, S	35.8 (33.4, 39.1)	35.8 (33.02, 39.25)	36 (33.8, 39)	0.498
D-D, μg/L	1.13 (0.69, 1.99)	1.21 (0.71, 1.98)	1.02 (0.6, 2)	0.062
**Invasive procedures**, ***n*** **(%)**				0.587
no	458 (71)	317 (70)	141 (72)	
yes	191 (29)	137 (30)	54 (28)	
**Tracheotomy**, ***n*** **(%)**				0.791
no	273 (42)	193 (43)	80 (41)	
yes	376 (58)	261 (57)	115 (59)	
**Nasogastric feeding**, ***n*** **(%)**				0.418
no	303 (47)	213 (47)	90 (46)	
yes	345 (53)	241 (53)	104 (53)	
BI, points	51 (33, 69)	52 (34, 69)	50 (32, 68)	0.718
GCS, points	8 (7, 9)	8.5 (7, 9)	8 (7, 9)	0.095
SSA, points	10 (7, 13)	10 (6.25, 13)	10 (7, 14)	0.395
Length of hospital stay, days	28 (21, 35)	28 (21, 36)	28 (21, 35)	0.303
Number of hospitalizations, times	3 (2, 4)	3 (2, 4)	3 (2, 4)	0.588

### Feature selection results

**Figure 2:**
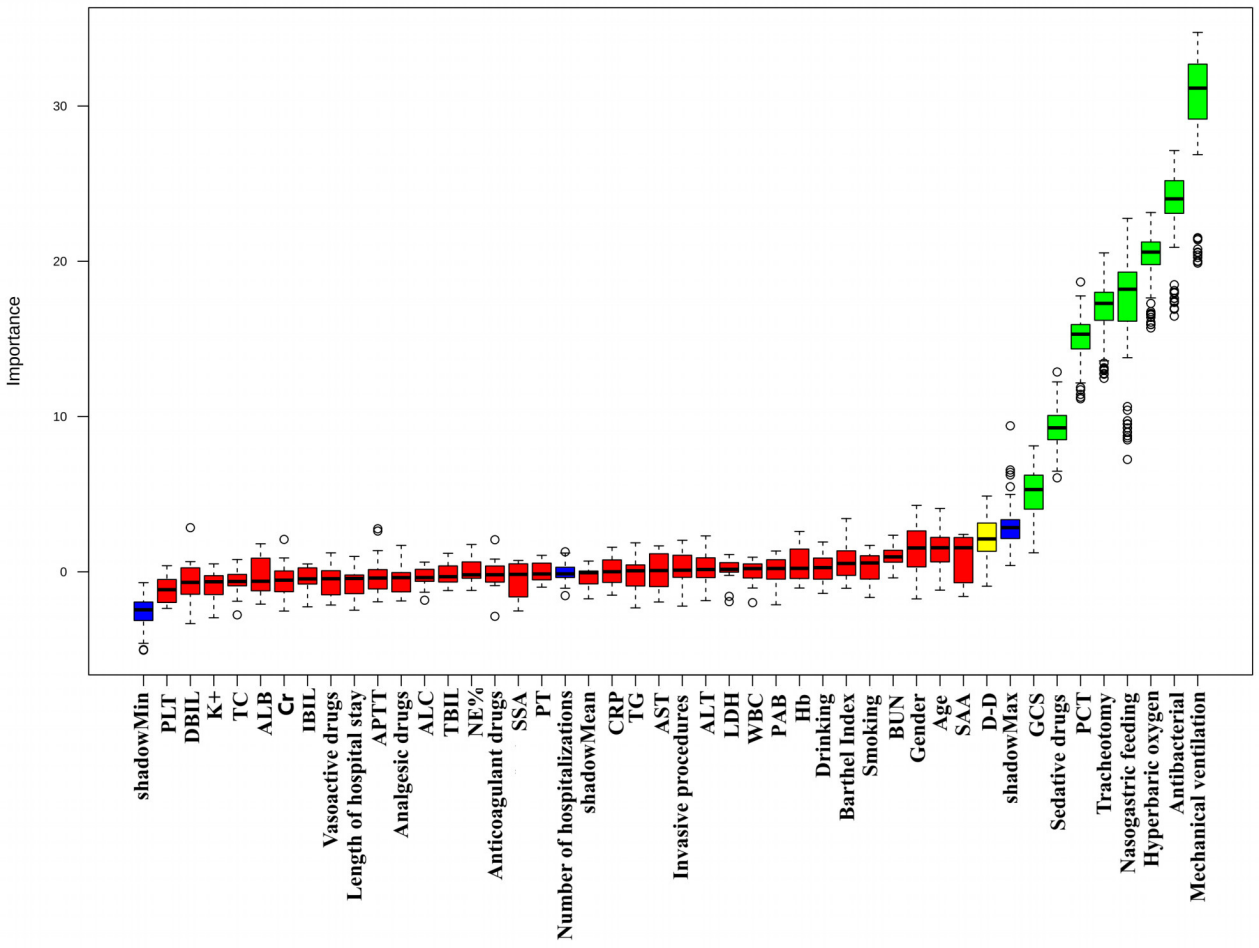
Feature selection analyzed by Boruta algorithm. The horizontal axis is the name of each variable, and the vertical axis is the Z-value of each variable. The box plot shows the Z-value of each variable in the model calculation. The green boxes represent the 8 important variables, the yellow represents tentative attributes, and the red represents unimportant variables.

To visually demonstrate the process and significance of feature selection, the Boruta algorithm identified eight key variables associated with the risk of PI in deep SICH patients. The identified variables include mechanical ventilation, antibacterial use, hyperbaric oxygen therapy, tracheotomy, sedative drugs, nasogastric feeding, the Glasgow Coma Scale (GCS), and procalcitonin (PCT), as presented in Figure 2.

### Model performance evaluation

**Figure 3:**
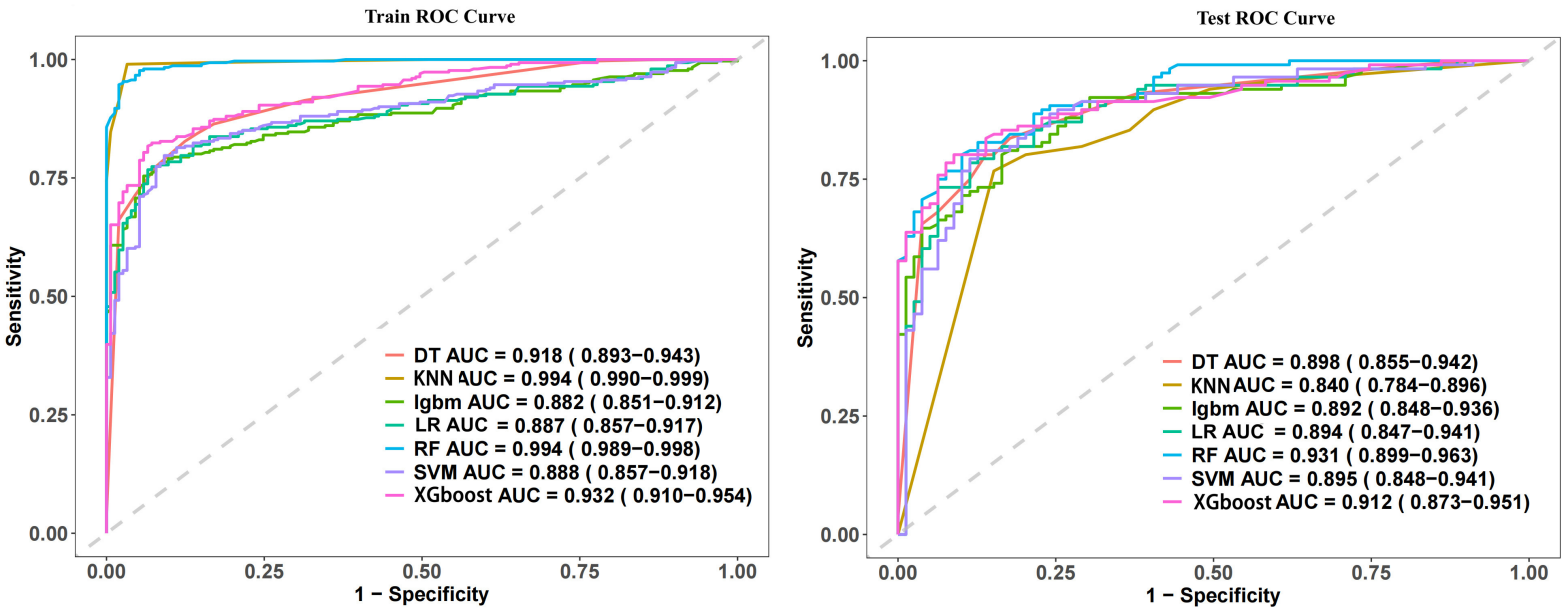
ROC Curve analysis of machine learning algorithms for predicting pulmonary infection in patients during the recovery phase of intracerebral hemorrhage.

This study assessed the performance of seven machine learning algorithms using metrics including ACC, SEN, SPE, PPV, and NPV, as summarized in Table 2. The AUC for each algorithm is presented in Figure 3. The RF model exhibited the best overall performance. In the training cohort, the ACC, SEN, SPE, PPV, and NPV were 0.9714, 0.9801, 0.9542, 0.9768, and 0.9605, respectively, achieving an AUC of 0.994 (95% CI: 0.989–0.998). In the testing cohort, the ACC, SEN, SPE, PPV, and NPV were 0.9554, 0.9619, 0.9362, 0.9453, and 0.9348, respectively, with an AUC of 0.931 (95% CI: 0.899–0.963. Pairwise DeLong tests were conducted to evaluate the statistical significance of AUC differences among the machine learning models in both the training and test sets (Table 3). In the training set, the Random Forest (RF) model demonstrated significantly higher AUCs than DT, LR, SVM, and LightGBM (all *p* < 0.01). No significant difference was found between RF and XGBoost (*p* = 0.13) or RF and KNN (*p* = 0.80).

**Table 2:** Performance evaluation of 7 machine learning algorithms on training and testing cohort.

**Model**	**Group**	**ACC**	**SEN**	**SPE**	**PPV**	**NPV**	**AUC (95% CI)**
LR	Train cohort	0.8260	0.7741	0.9281	0.9549	0.6762	0.887 (0.857–0.917)
	Test cohort	0.8256	0.8190	0.8354	0.8796	0.7586	0.894 (0.847–0.941)
DT	Train cohort	0.8524	0.9091	0.7560	0.8638	0.8301	0.918 (0.893–0.943)
	Test cohort	0.8308	0.8739	0.7738	0.8362	0.8228	0.898 (0.855–0.942)
**RF**	**Train cohort**	**0.9714**	**0.9801**	**0.9542**	**0.9768**	**0.9605**	**0.994 (0.989–0.998)**
	**Test cohort**	**0.9554**	**0.9619**	**0.9362**	**0.9453**	**0.9348**	**0.931 (0.899–0.963)**
XGboost	Train cohort	0.8855	0.8704	0.915	0.9527	0.7821	0.932 (0.910–0.954)
	Test cohort	0.8308	0.8621	0.7848	0.8547	0.7949	0.912 (0.873–0.951)
SVM	Train cohort	0.1652	0.2027	0.092	0.3050	0.055	0.888 (0.857–0.918)
	Test cohort	0.1795	0.1638	0.2025	0.2317	0.1416	0.895 (0.848–0.941)
KNN	Train cohort	0.9656	0.9090	0.9367	0.9358	0.9108	0.994 (0.990–0.999)
	Test cohort	0.7641	0.8534	0.6329	0.7734	0.7463	0.840 (0.784–0.896)
LightGBM	Train cohort	0.8261	0.7641	0.9346	0.9583	0.6682	0.882 (0.851–0.912)
	Test cohort	0.8051	0.7845	0.8354	0.8750	0.7253	0.892 (0.858–0.936)

Bold indicates the best-performing metric among all models.

**Table 3:** Pairwise delong test *p*-values for auc comparisons among the seven machine learning models in the training and testing cohort.

**Model**	**Train set** ***P*****-values**							**Test set** ***P*****-values**						

	**DT**	**LR**	**RF**	**XGboost**	**SVM**	**KNN**	**LightGBM**	**DT**	**LR**	**RF**	**XGboost**	**SVM**	**KNN**	**LightGBM**
DT	1	0.12	**0.00**	0.00	0.01	0.00	0.10	1	0.89	**0.05**	0.06	0.86	0.01	0.86
LR		1	**0.00**	0.00	0.98	0.00	0.94		1	0.22	0.22	0.99	0.15	0.98
RF			1	0.13	**0.00**	0.80	**0.00**			1	0.95	**0.02**	**0.00**	0.19
XGboost				1	0.00	0.00	0.00				1	0.06	0.00	0.19
SVM					1	0.00	0.92					1	0.03	0.96
KNN						1	0.00						1	0.14
LightGBM							1							1

Bold indicates statistically significant AUC differences (*p* < 0.05) compared to the RF model based on DeLong's test.

In the test set, RF achieved the highest AUC (0.931). Statistically significant differences were observed between RF and SVM (*p* = 0.02), KNN (*p* = 0.00), and DT (*p* = 0.05). Differences between RF and LR (*p* = 0.22), XGBoost (*p* = 0.95), and LightGBM (*p* = 0.19) were not statistically significant, suggesting similar performance among these models in external validation.

### Visualization by SHAP

**Figure 4:**
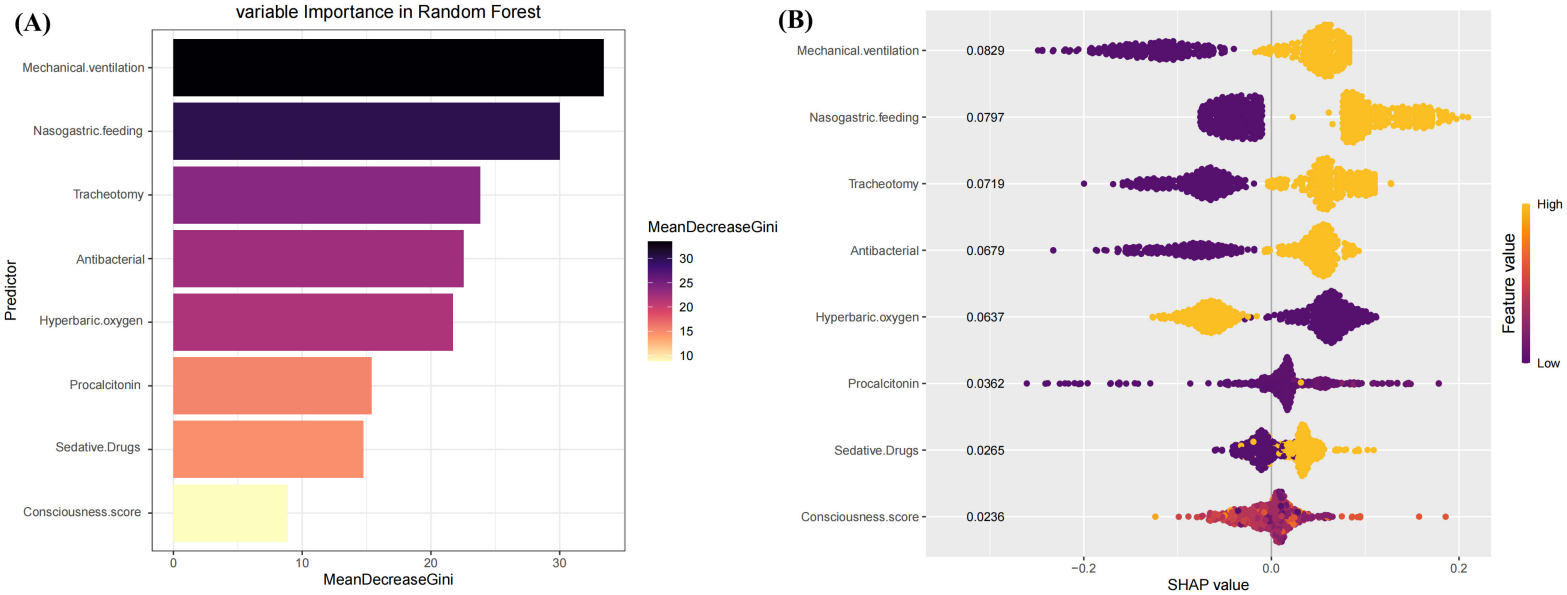
Analysis of variable importance and SHAP values in the random forest model. **(A)** The horizontal axis represents the average decrease in the Gini coefficient. In the RF model, the top 8 variables most associated with pulmonary infection are ranked by importance. A larger decrease in the Gini coefficient indicates a higher importance of the variable in the model. **(B)** This is a SHAP plot illustrating the impact of each feature variable on the risk of pulmonary infection.

As shown in Figure 4A, the ranking of feature variables influencing PI risk, based on the mean decrease in the Gini coefficient, is as follows: mechanical ventilation, nasogastric feeding, tracheotomy, antibacterial use, hyperbaric oxygen therapy, procalcitonin levels, sedative drug use, and consciousness score. Figure 4B illustrates the influence of these feature variables on the risk of PI. Mechanical ventilation, nasogastric feeding, tracheotomy, antibacterial use, sedative drug use, and elevated procalcitonin levels were found to significantly increase the risk of PI. Moreover, patients with higher consciousness scores or those receiving hyperbaric oxygen therapy exhibited a reduced risk of PI.

## Discussion

In this study, the Boruta algorithm was employed for robust feature selection, and a risk prediction model for pulmonary infection (PI) during the recovery phase of deep subcortical intracerebral hemorrhage (deep SICH) was developed using seven machine learning algorithms. Among these, the random forest (RF) model demonstrated the highest predictive performance, with AUCs of 0.994 (95% CI: 0.989–0.998)in the training set and 0.931 (95% CI: 0.899–0.963) in the test set, along with superior accuracy, sensitivity, specificity, positive predictive value (PPV), and negative predictive value (NPV). To further evaluate the statistical significance of performance differences between models, pairwise DeLong tests were conducted based on AUC values. In the training cohort, the RF model exhibited significantly better discrimination compared to DT, LR, SVM, and LightGBM (all *p* < 0.05), while showing no significant difference vs. KNN and XGBoost. In the test cohort, although the differences were not statistically significant across most comparisons, RF maintained the highest overall classification performance. The superior robustness of RF can be attributed to its ensemble architecture, which mitigates variance by integrating multiple decorrelated decision trees. This structure enhances its ability to capture complex non-linear interactions and reduces overfitting, particularly in multidimensional, noise-prone clinical datasets. These advantages affirm RF as the most reliable and generalizable model in our setting, offering strong potential for clinical translation. SHAP (Shapley Additive Explanations) analysis further elucidated the relative contribution of each variable in PI prediction, ranking their influence from greatest to least as follows: mechanical ventilation, nasogastric feeding, tracheotomy, antibacterial drug use, hyperbaric oxygen therapy, elevated procalcitonin levels, sedative drug use, and consciousness status.

### Analysis of key variables and mechanisms

The final eight variables retained in our model were selected through an integrative approach combining statistical rigor and clinical reasoning. Initially, the Boruta algorithm identified a set of statistically significant predictors based on their relevance to the outcome. Among these, eight variables were ultimately selected based on two primary criteria: (1) their high importance scores in the random forest model, and (2) well-established clinical evidence linking them to pulmonary infection. This dual-criterion strategy ensured that all retained predictors not only contributed to model performance but also had sound medical justification. The following section presents detailed pathophysiological mechanisms and literature support for each key variable.

This finding is consistent with previous studies (27, 28), which have identified mechanical ventilation as a major contributor to hospital-acquired pneumonia (HAP) and ventilator-associated pneumonia (VAP) due to its complex and multifaceted underlying mechanisms. Research suggests that mechanical ventilation (29), particularly via endotracheal intubation, compromises the airway's mechanical barriers, thereby facilitating the invasion of external pathogens into the lower respiratory tract. Patients undergoing prolonged mechanical ventilation often develop airway secretion accumulation, which, due to altered pressure dynamics and pathological conditions, becomes increasingly difficult to clear. This condition creates an ideal environment for bacterial colonization and proliferation, particularly for Gram-negative pathogens such as *Pseudomonas aeruginosa* (30) and multidrug-resistant strains like Methicillin-resistant *Staphylococcus aureus* (MRSA) (31). Furthermore, mechanical ventilation may disrupt alveolar gas exchange and alter local pressure dynamics, resulting in hypoperfusion or alveolar overdistension. These pathological alterations can cause alveolar epithelial cell injury and induce inflammatory responses, thereby heightening the risk of infection. Studies have also demonstrated that pressure changes induced by mechanical ventilation can modify the pulmonary microenvironment, further increasing the likelihood of infection (32). The findings of this study suggest that in clinical practice, enhanced interventions should be implemented for mechanically ventilated patients to reduce the incidence of PI in deep SICH. Strict adherence to VAP prevention measures is recommended, including routine airway clearance, optimization of ventilation parameters, and early extubation.

A meta-analysis revealed that nasogastric tube feeding could increase the risk of PI in SICH patients by 9.87 times (24). This study, through a random forest model, identified nasogastric tube feeding as the second most significant factor influencing the risk of PI, a correlation further validated in specific samples through SHAP value analysis. The prolonged presence of a nasogastric tube in the nasopharyngeal region may alter the local microenvironment, providing favorable conditions for bacterial colonization. Existing research has demonstrated that bacterial colonization in the nasopharynx is a significant source of respiratory infections, and the long-term retention of a nasogastric tube significantly increases colonization rates (33). Additionally, when gastric acid secretion is suppressed in patients receiving nasogastric tube feeding (e.g., through the use of proton pump inhibitors (PPIs) or H_2_ receptor antagonists), the bactericidal effect of gastric acid is weakened, potentially leading to excessive growth of gastrointestinal bacteria. Some pathogens may even enter the lungs via the “gut-lung axis,” triggering infections. Studies have also found a significant increase in the number of Gram-negative bacteria commonly found in gastric cultures of patients receiving nasogastric tube feeding, and these bacteria are closely related to respiratory infection pathogens (34). Furthermore, contamination of nasogastric tube feeding solutions is closely associated with infection risk, as contaminated feeding solutions may directly introduce pathogens into the gastrointestinal or respiratory tracts, leading to secondary infections (35). Moreover, the long-term mechanical irritation of the nasal and pharyngeal mucosa by the nasogastric tube may cause local inflammation, weaken the mucosal barrier, and impair local immune function, thereby promoting pathogen invasion into the respiratory tract (36). In summary, while nasogastric tube feeding is a necessary method of nutritional support, it carries high risks. Targeted nursing measures, such as elevating the head of the bed, enhancing aspiration risk assessment, and appropriately adjusting feeding speed, are essential to effectively reduce the risk of infections associated with nasogastric tube feeding.

The RF model ranked tracheotomy as the third most critical determinant of PI risk. SHAP analysis further validated that tracheotomy substantially increases the risk of pulmonary infection, a finding consistent with previous studies (37). Tracheotomy compromises the airway's natural barrier function, facilitating pathogen invasion into the lower respiratory tract (38). Moreover, the accumulation of airway secretions following tracheotomy fosters an environment conducive to bacterial proliferation and biofilm formation, further increasing the risk of infection (39). Therefore, rigorous nursing management is crucial for tracheotomy patients, including regular tracheostomy tube replacement, effective humidification therapy, and continuous monitoring of microbial colonization to mitigate infection risk. Procalcitonin (PCT) is a crucial biomarker for bacterial infections and disease severity and has been extensively applied in clinical practice (40). Lu et al. confirmed that PCT is a key biomarker of bacterial infection, with elevated levels frequently indicating exacerbated inflammatory responses, especially in patients with pulmonary infections (41). Wang et al. demonstrated that dynamic monitoring of PCT levels facilitates early identification of high-risk infection patients and provides guidance for the rational use of antibiotics (42). Similarly, this study identified PCT levels as a key determinant of PI risk during the recovery phase in patients with deep SICH. SHAP value analysis demonstrated a strong correlation between elevated PCT levels and an increased risk of PI. These findings underscore the necessity of monitoring and regulating PCT levels in the clinical management of deep SICH to mitigate PI risk and optimize clinical decision-making.

The RF model also identified antibacterial and sedative drugs as significant determinants of PI risk. SHAP analysis revealed that broad-spectrum or frequent antibiotic use substantially increases infection risk, consistent with findings from previous studies (23, 43, 44). Excessive antibiotic use can promote the emergence of resistant strains, such as multidrug-resistant Gram-negative bacteria (MDR-GNB), thereby complicating pulmonary infections (45). Inappropriate treatment or prolonged exposure to an antibiotic-rich environment may disrupt the patient's normal microbiota, further increasing infection risk (46). Therefore, rational antibiotic use is crucial, emphasizing targeted therapy guided by microbial culture and susceptibility testing, implementation of antimicrobial stewardship programs, and adoption of short-course regimens to minimize the risk of antibiotic-associated infections. Beyond antibiotics, SHAP analysis further revealed that sedative drugs substantially elevate the risk of PI. Studies have demonstrated that ICU patients undergoing prolonged benzodiazepine therapy exhibit a significantly higher risk of developing respiratory infections (47). The underlying mechanisms include suppression of the cough reflex and impairment of ciliary movement by sedative drugs, resulting in airway secretion retention (48). This condition fosters an environment conducive to pathogen proliferation, thereby heightening infection risk. Sedative drugs can compromise swallowing reflexes and facilitate aspiration, further predisposing patients to PI (49). The synergistic effects of impaired airway clearance and increased aspiration risk establish sedative use as a pivotal contributor to PI development. To mitigate the infection risk associated with sedative drugs, precise sedation management is essential. Key strategies involve dynamic titration of sedative dosages based on patient conditions, optimized airway management to ensure effective secretion clearance, and preventive measures to reduce aspiration risk. Non-pharmacological interventions, such as positional therapy and minimizing sedative use when clinically feasible, offer additional strategies to reduce these risks.

Consciousness score assessments are widely acknowledged as an independent and crucial risk factor for PI in stroke patients (14). In this study, although consciousness score assessments were identified as a significant factor influencing PI risk, their relative impact during the recovery phase of deep SICH was lower than that of other factors. SHAP value analysis further confirmed that lower consciousness score assessments, indicative of poorer consciousness evaluation results, significantly increased PI risk. Previous studies (50) have shown that patients with reduced consciousness often experience impaired respiratory defense mechanisms, including diminished cough reflexes and the retention of airway secretions, which collectively heighten susceptibility to pathogen-induced infections. This study highlights the necessity of proactive airway management for patients with low consciousness score assessments. Regular sputum clearance, maintaining airway patency, and preventing secretion accumulation are vital measures to mitigate infection risk. Implementing neurorehabilitation strategies and other interventions aimed at improving consciousness score outcomes can facilitate central nervous system recovery while also significantly reducing PI incidence.

HBOT, an emerging therapeutic approach, demonstrated a significant protective effect against PI in patients recovering from SICH, as revealed by SHAP analysis. Although previous literature has rarely reported this association, emerging evidence from experimental and clinical studies provides a biologically plausible rationale. The potential protective mechanisms of HBOT in infection control may be summarized as follows: first, HBOT markedly increases tissue oxygen tension, which inhibits the growth of anaerobic bacteria and enhances the efficacy of certain antibiotics (51). Second, HBOT directly inhibits anaerobic bacteria while enhancing the bactericidal activity of immune cells, such as neutrophils and macrophages, thereby strengthening host defense against infections (52). Additionally, HBOT suppresses excessive inflammatory mediator release, reduces tissue edema and inflammation, and mitigates lung tissue damage caused by infections (53). Finally, HBOT promotes endothelial cell proliferation, enhances angiogenesis, and accelerates wound healing, thereby supporting recovery at the infection site (54). These mechanisms are consistent with recent findings from experimental and clinical studies exploring the immunomodulatory and antimicrobial effects of HBOT in critical care settings (55, 56). The findings highlight HBOT's critical role in alleviating local tissue hypoxia and reducing PI incidence through multiple synergistic mechanisms. Compared with traditional risk factors, HBOT exhibited particularly strong protective effects in this model. These insights provide a robust theoretical foundation for the clinical application of HBOT, particularly in the management of high-risk deep SICH patients, and underscore its potential value for future research and clinical exploration.

### Clinical implications and application value

This study integrates a random forest model with SHAP interpretability analysis to develop an accurate tool for assessing PI risk in patients recovering from deep SICH. The model effectively identifies high-risk patients, providing a robust scientific foundation for clinical interventions. For instance, optimizing airway management and dynamically monitoring infection markers are recommended for patients receiving mechanical ventilation or enteral feeding. Similarly, integrating model predictions for patients with elevated PCT levels can help optimize antimicrobial therapy strategies. Integrating SHAP analysis enhances the model's interpretability, enabling clinicians to intuitively comprehend the specific impact of risk factors and providing critical guidance for personalized treatment. The findings of this study contribute to more efficient early identification of PI, improved patient outcomes, and optimized allocation of healthcare resources.

### Innovations and limitations of the study

This study presents several innovations. To our knowledge, it is the first to focus specifically on the risk of PIin patients during the recovery phase of deep SICH. The use of a RF model combined with SHAP analysis enabled high-precision risk prediction while preserving interpretability. Notably, the model identified HBOT as a potential protective factor, offering a novel insight into infection management for this population. Importantly, given the retrospective nature of the study, data completeness was strictly controlled. From an initial dataset of 1,021 patients, 649 cases with complete clinical information were retained after standardized exclusion criteria. Missing data were assessed using SPSS frequency analysis, confirming that the final dataset was complete. This rigorous approach minimized the risk of information bias and enhanced the validity of the model. Nevertheless, several limitations should be acknowledged. First, the single-center design and limited sample size may restrict the external applicability of the model, necessitating validation through multi-center studies with larger cohorts to enhance robustness. Second, the model relies solely on static baseline data, lacking integration of time-series data, such as dynamic changes in PCT levels, which may reduce sensitivity to disease fluctuations. Third, although the model highlights the significance of HBOT, its specific mechanisms and intervention effects require further clinical trials for validation. Fourth, the study did not include prospective evaluation of model-guided interventions, and thus the practical impact and causal pathways of the identified predictors remain to be verified in randomized controlled settings. Additionally, the exclusion of intraventricular extension of cerebral hematoma—a well-established predictor of poor early outcomes—represents another potential limitation. As highlighted by Arboix et al. (57), hemorrhage extending into the ventricles markedly increases early mortality and worsens neurological recovery. Although this variable was omitted due to dataset limitations and the study's focus on pulmonary complications rather than mortality, its clinical importance is evident. Future research should incorporate this parameter to enhance prognostic precision and enrich the pathophysiological understanding of infection risk in deep SICH. Finally, this study did not perform hyperparameter tuning during model development. All machine learning algorithms were implemented using default or empirically defined parameters. Although the use of 10-fold cross-validation with five repetitions helped mitigate overfitting, the absence of systematic parameter optimization may have constrained model generalizability. Future studies should incorporate grid search or Bayesian optimization to refine model performance and robustness.

### Future directions

In addition to addressing the limitations identified above, future research should also aim to construct multicenter prospective cohorts to externally validate the predictive model and assess its generalizability across diverse populations. Integration of dynamic clinical variables—such as serial procalcitonin levels, real-time infection markers, and temporal consciousness fluctuations—may improve the model's sensitivity and adaptability. Furthermore, interventional studies are warranted to evaluate the effectiveness of individualized preventive strategies guided by model-based risk stratification. Exploring the underlying pathophysiological mechanisms linking key risk factors (e.g., sedation, hyperbaric oxygen therapy) to pulmonary infection may also enhance the translational value of predictive modeling in post-ICH care.

## Conclusions

This study presents a novel and clinically interpretable machine learning approach for predicting pulmonary infection risk in patients during the recovery phase of deep subcortical intracerebral hemorrhage (deep SICH). By integrating a high-performing random forest model with SHAP analysis, we established a robust predictive framework capable of identifying high-risk individuals with exceptional precision. Beyond predictive accuracy, the model's interpretability facilitates personalized clinical decision-making, supporting targeted interventions such as airway optimization, timely infection surveillance, and rational antibiotic use. These findings not only offer a practical tool for improving patient outcomes and reducing healthcare burden, but also underscore the value of explainable artificial intelligence in bridging the gap between complex data modeling and real-world clinical application. Future extensions of this work may further enhance its translational potential and contribute to more precise, individualized care pathways in neurocritical rehabilitation.

## Data Availability

The raw data supporting the conclusions of this article will be made available by the authors, without undue reservation.
